# Building an evaluation infrastructure to capture process and progress within a Clinical and Translational Science Awards hub

**DOI:** 10.1017/cts.2025.10047

**Published:** 2025-06-09

**Authors:** Maggie Padek, Dinesh Pal Mudaranthakam, Sam Pepper, Mary Penne Mays, Shellie D. Ellis

**Affiliations:** 1 Frontiers Clinical and Translational Science Institute, University of Kansas Medical Center, Kansas City, KS, USA; 2 Department of Biostatistics & Data Science, University of Kansas Medical Center, Kansas City, KS, USA; 3 Research Informatics, Research Administration, University of Kansas Medical Center, Kansas City, KS, USA; 4 Department of Population Health Kansas University Medical Center, Kansas City, KS, USA

**Keywords:** Evaluation, REDCap, logic models, data transparency, Clinical and Translational Science Awards operations

## Abstract

To improve its management capacity, Frontiers Clinical and Translational Science Institute overhauled its evaluation infrastructure to be comprehensive, efficient, and transparent in demonstrating outputs and outcomes. We built a platform that standardized measures across program areas, integrated continuous improvement processes, and reduced the data entry burden for investigators. Using the Utilization-Focused Evaluation Framework, we created logic models to identify appropriate metrics. We built the evaluation data platform within REDCap to capture requests, events, attendance, and outcomes and to push work processes to Navigators. We initiated a membership model to serve as the backbone of the platform which allowed tailored communication, demographic data capture, and reduced data entry burden. The platform consists of nine REDCap projects across multiple programmatic areas. Using REDCap Dynamic SQL query fields and External Modules, the membership module was integrated into all forms to check and collect membership before service access. Data is synched to a dashboard for tracking outputs and outcomes in real-time. Since the launch of the evaluation platform in Fall 2022, Frontiers has increased its workflow efficiency and streamlined continuous improvement communication. The platform can serve as a model for other hubs to build efficient processes to create comprehensive and transparent evaluation plans.

## Background

Evaluation and management of large-scale programs and institutes can prove demanding, especially with limited financial resources and staff capacity. Infrastructure grants and institutes can be particularly challenging to evaluate, given the size, funding, activities, and goals that make up their structure [1]. For example, Clinical and Translational Science Awards (CTSA) pose unique difficulties in aligning evaluation data management across the breadth of activities undertaken [2,3]. Aligning evaluation plans, data collection processes, and the platforms on which they operate is just as critical as creating effective evaluation plans. Creating cost effective, accessible, centralized, and comprehensive systems may address these challenges.

The Frontiers Clinical and Translational Science Institute (CTSI) is the primary driver of institutional research infrastructure in a consortium of research institutions in the Midwest United States. It is supported by a $25 million dollar grant from the National Center for Advancing Translational Science and is comprised of eight academic and medical partner institutes: University of Kansas Medical Centers, University of Kansas, University of Kansas Health System, Children’s Mercy Hospital Kansas City, University of Missouri-Kansas City, Kansas City University, Kansas State University, and Saint Luke’s Health System. Before Frontiers 2022 grant renewal, evaluation was based on a case study approach [4] and there was no active internal, centralized, and standardized infrastructure dedicated to evaluating processes, outcomes, and impacts, reflecting NIH’s updated expectations [5].

Frontiers prioritized implementing a system that could capture evaluation data across several different outcome and impact metrics, required minimal regular manual maintenance, was accessible to staff across multiple research institutions with independent IT infrastructures and could be adaptable to leadership and funders priorities. Given that many CTSA hubs are now partnering with multiple research institutions, independent from the grant-holding institution [6], there is a need for evaluation data capture processes and infrastructure that can be adaptable to different organizational workflows as well as technologically accessible. Many established software programs can become costly when making multiple customizations and only allow the license holder (often the grant-holding institution) to determine which metrics should be captured.

To address recommendations derived from the CTSA Evaluation Guidelines Working Group, we undertook the initiative to create an evaluation approach in a manner that would build infrastructure and capacity, utilize existing resources, data, and workflow processes, and, ultimately, convey the impact of the extensive work of our institute [5]. Given that Frontiers operates across eight different partner institutions, provides services across thirteen specified “cores” and covers a catchment area of almost 109,000 mi^2^ with almost 100 support staff involved in day-to-day operations, our evaluation team needed a system that was efficient and accessible to optimize the workflow and capture relevant outputs and outcomes data at the point of service.

While many established software systems exist for data capture utilized by other CTSAs [3,7], none seemed to accommodate our institute’s customization needs and accessibility concerns. We believe this work is novel as there has been little documentation in the literature on how evaluation systems can be built within a CTSA hub and how one can harness established resources (such as REDCap) to do so more cost-efficiently. We sought to describe the features and design considerations of our efforts and the subsequent productivity outputs to inform similar evaluation infrastructure efforts.

## Framework & approach

To design this new infrastructure, our team employed a Utilization-Focused framework [8] to guide the process of building a platform that was of use to all program areas within the hub and could be adaptable to already established workflows and future data needs. Initial meetings were held with representatives from informatics, biostatistics, administration, and leadership teams to outline data capture instruments and data flow to meet both internal and external reporting standards. The evaluation team then applied logic models to each core programmatic area to identify the specific metrics, outcomes, and impacts that related to their specific aims. The evaluation team engaged directly with each cores’ leadership in this iterative process to gain leadership awareness for the need of evaluation processes as well as gain buy-in for the new infrastructure. This process aligned with the principles of the Utilization-focused framework by ensuring stakeholder engagement, designing for relevance, and maintaining credibility for the infrastructure, and building capacity for stakeholders to utilize the platform [8]. We use administrative, process, and qualitative data to describe the evolution and feasibility of our evaluation platform.

**Figure 1.**
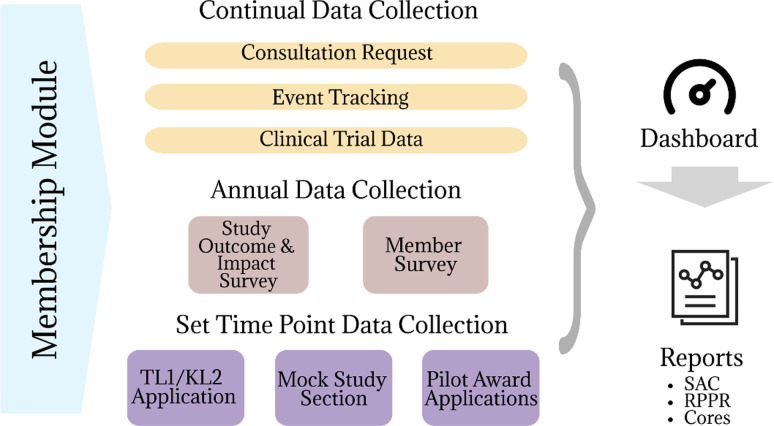
Evaluation platform framework.

Once all activities, outputs, and outcomes were finalized in each Core’s logic model, we categorized each activity to better understand the data sources to derive these outputs. Categories included educational activities, events, mentoring, consultation, connection, funding, research tools, organizational changes, training programs, sustainability activities, communications, and partnership engagement. This categorization served as a framework to simplify and align activities across Cores. From there we identified the unit of analysis (e.g., investigator, study, event) and proximity of the outcome (e.g., short-term, or long-term) to identify where and how data collection should take place. Finally, we created an inventory to determine which data sources were already established and which ones needed to be built based on conversations with programmatic leadership. The missing variables were the basis of an Evaluation Platform (EP) (Figure 1) fed by a series of surveys.

Once the logic models were established, they were reviewed with each Core bi-annually. An evaluation team member attends Core leadership meetings and reviews the progress of each Core’s activities. Relevant edits are noted within a primary logic model, and any progress toward objectives is documented. Cores receive a summary report of their progress each December to help draft their annual NIH progress report (RPPR). The evaluation team holds the primary copy of the logic models but sends updated versions on request.

### Informatics collaboration

Initially, leadership explored several software options for building the EP but decided to use REDCap [9,10]. Leadership’s decision was based on leveraging existing institutional REDCap expertise and champions. REDCap was also chosen to further the research capacity throughout Frontiers by familiarizing Frontiers-supported research staff to the functionality and advanced capabilities of REDCap. Ease of access and user familiarity, and its compliance with several IT standards and HIPAA also weighed into the decision.

### Membership

In conjunction with the revised evaluation plan, the Frontiers leadership team decided to transition to a membership model at the beginning of the new funding cycle, in line with many CTSA hubs. The Frontiers Informatics team assisted in the development of a membership form to capture contact information, communication preferences, career interests, and personal demographics about the individual investigator, community partner, or staff member (Appendix A). The membership form serves as the backbone for the EP. With assistance from our REDCap Administrator [9,10] the form was connected to several other projects through REDCap Dynamic SQL [11], query fields and External Modules [12]. Additional guidance on technical aspects of these developments can be found at the referenced GITHub site. The consultation request, pilot applications, KL2 and TL1 applications, and Mock Study Section applications all have the membership form embedded to ensure individuals are registered members before they request services.

This integration also assists with tracking demographic characteristics of Frontiers members more readily. Membership is free and requires only the one-time completion of the REDCap form. Once demographic information is entered into the Frontiers database, it does not have to be entered again but can be updated as needed. While this work has been deemed non-human subjects research and does not require IRB oversight, the Evaluation team has applied the same data privacy standards for member data as it would for other human subjects-related research. All identifiable data is stored in REDCap which as noted is HIPAA and PHI compliant. Access to the specific REDCap projects is limited to the evaluation team, all who have completed CITI training. Any identifiable data is stored on local HIPAA & PHI compliant servers. Demographic data is only shared in the aggregate and any necessary individual data is deidentified.

### Consultation form

The consultation form has the dual purpose of connecting members to requested services and resources and serving as a platform for Frontiers Navigators to document their efforts and service usage. Navigators are staff representatives for each service area the CTSI supports, and they are meant to serve as the connection point for members to access services by either performing the request themselves or directing members to the appropriate investigator or resource that can address their needs.

A list of all the Cores and their relevant resources is included in the consultation form. User-friendly language ensures that investigators recognize resources they might need, as the grant-derived core labels do not always describe the resources aptly. Within REDCap, branching logic helps users establish the specificity of the service they require. Each service selection is then linked to the appropriate Navigator through an email alert within REDCap. Navigators are trained on how to respond to individual requests and record their interactions and consultation results within the platform.

### Workflow surveys

The consultation request form is linked to a series of subsequent “surveys,” directed to the Navigator, which allows the Navigator to document consultation scheduling and disposition. All navigator documentation is programed for email delivery based on the consultation request date and includes a series of reminders to ensure the timely completion of the consultations. Navigators can also enter a consult request on behalf of someone to document their work or if someone has reached out to them directly. After the Navigator marks the consultation complete, a follow-up satisfaction survey is automatically sent to the user through REDCap to assess the quality of services. Completion of the consultation service also triggers a one-year follow-up survey sent through REDCap to assess any long-term products and impacts coming from the consultation.

### Event form

In addition to consultations, Frontiers tracks attendance at the events they sponsor. Unlike the consultation form series, internal staff exclusively uses this series. Staff or Navigators enter basic information (date, time, title, audience, format, etc.) about the event into the REDCap form as soon as basic details are confirmed. When a new record is created in this form, an email alert is sent to the Communications Coordinator to let them know to connect with the sponsoring Core. The Communications Coordinator will then work with that Navigator to determine the most appropriate way to advertise the event through the Frontiers network. Although staff can retrospectively enter event information, it is encouraged that they enter the information before the event so that appropriate marketing and communications can occur.

Once the event occurs, an automated email is pushed via REDCap to the designated point of contact to collect attendance data. Navigators are encouraged to capture names and email addresses at all events but if not feasible (e.g., health fairs), aggregate attendance numbers can be entered. A template spreadsheet is provided for attendance names and emails for the navigators to upload within the REDCap form. Once event sponsors record attendance, an email is sent to the evaluation team alerting them of a new attendance report. Attendance reports are reformatted and uploaded in a second REDCap project where event satisfaction surveys are triggered, and demographic information of Frontiers member attendees can be pulled from the membership form.

### Annual data collection instruments

Finally, we established annual data collection instruments to fill the remaining data gaps. An annual member assessment is sent out at the conclusion of the grant year to conduct a process evaluation of the services and operations occurring within Frontiers and a needs assessment of what resources and services are still desired by members. A set of base questions is collected annually, but Cores can modify items each year to capture new issues that arise. Questions regarding members’ perceived benefits from Frontiers and their perceived level of influence and trust [13,14] with the Frontiers CTSI assess changes in the Institutes’ responsiveness and partnership with its individuals’ members year-to-year. Open-ended qualitative questions are utilized during the annual member survey to more holistically capture how Frontiers has impacted the members research capacity or their projects’ outcomes.

Additionally, a Project Outcomes and Impact survey is sent annually to past pilot awardees and trainees for long-term follow-up of any *study* outcomes. Surveys are sent to awardees at least one year after Frontiers support ends to capture any residual activity or impacts related to their funding awards.

### Evaluation dashboard

In collaboration with the Biostatistics & Informatics team, we created a dashboard using R Shiny that displays summaries of the data collected from each REDCap project. This is done using dynamic tables, charts, and figures with user-selected filtering capabilities. The dashboard is hosted using Posit’s Shiny Server application which is accessible using an internal URL. The initial phase of the dashboard pulled data daily from the membership form, consultations form, event forms, and study accrual data from internal Clinical Research Information Systems. The second phase incorporated quarterly participant data from the Clinical Trials Service Units and the Informatics and Biostatistics, Epidemiology, Research Design Core as they had well established workflows recorded in separate REDCap forms. Rather than requiring duplication of work, integrating these forms in the third-party database created an automated process for reporting. The goal of the dashboard is to provide data transparency for the internal cores and to provide basic data feedback in assistance with as-needed reports. To maintain privacy, data is structured to only report variables in the aggregate when *n* > 11.

## Results

### Logic models

In aligning with concepts of the Utilization-Focused framework [8], our logic models serve as a tool to enhance bidirectional communications by conduct regular check-ins with our core leadership, refine activities and metrics based on current progress and identify which EP data sources can be used to document these outcomes. Each Spring, we reviewed logic models with each core to determine the accuracy of the activities and stated outcomes. We took notes during team meetings throughout the year and entered them into the master version of the spreadsheet for progress tracking. Based on these notes, we implemented progress reviews in each Fall and final updates were noted in a report for each core along with their RPPR preparation materials in December. We provided aggregated data on each cores’ consultations recorded and demographics of those who received services and/or attended events sponsored by the cores. Core leads provided verbal feedback that reports were useful for their preparations and tying their activities to stated outcomes within the logic models.

### Membership

The Communications Specialist utilized an older listserv to inform previous Frontiers stakeholders that the institute would be transitioning to a membership model and that the listserv would be discontinued after a set time point. The request was for all former partners and stakeholders to register as members to ensure their communications from Frontiers would continue. The membership format launched in September 2022, and the Communications Specialists discontinued the use of the old listserv at the end of the grant year (June 2023). At that point, there were over 400 members in the Frontiers member database, and as of June 2024, there are more than 700 members. Table 1 shows a snapshot of Frontiers Membership demographics at the end of year 1 and 2. The Communication Specialist updated contact lists weekly based on Members’ stated communication preferences from their membership forms. An evaluation of the integration of the membership form within the funding and training award applications and the Mock Study Section application is in progress.

**Table 1.** Snapshot of Frontiers Membership data at end of year 1 & year 2 (June 2023 & 2024)

	Year 1 (n = 416)*	Year 2 (n = 750)*
**Career Stage^**	**% (n)**	**% (n)**
New Investigator	35.4% (130)	34.2% (235)
Experienced Investigator	23.3% (85)	17.7% (122)
Trainee (Undergraduate, Graduate student, Resident, Post-doctoral)	14.7% (56)	16.1% (121)
Other (includes research staff)	26.1% (96)	27.8% (209)
**Institution** ^**#**^	**%(n)**	**%(n)**
University of Kansas Medical Centers (Kansas City, Wichita, Salina)	52.8% (239)	52.7% (429)
Children’s Mercy Hospital of Kansas City	17.7% (80)	15.1% (123)
University of Missouri-Kansas City	8.8% (40)	8.5% (69)
University of Kansas-Lawrence	5.7% (26)	7.1% (58)
University of Kansas Health System	4.6% (21)	5.6% (46)
Kansas City University	3.5% (16)	3.1% (25)
Kansas State University	1.5% (7)	2.3% (19)
Saint Luke’s Health System	1.3% (6)	1.2% (10)
**Area of Research**	**% (n)**	**% (n)**
Basic Research	13.7% (57)	15.8% (119)
Pre-Clinical Research	7.2% (30)	7.3% (55)
Clinical Research	38.8% (161)	45% (337)
Implementation Research	10.4% (43)	7.6% (56)
Population-Based Research	12.8% (53)	10.5% (79)
Not Applicable	17.1% (71)	13.7% (103)
**Gender Identity^**	**% (n)**	**% (n)**
Woman	60.1% (214)	60.9% (412)
Man	36.5% (130)	35% (237)
Transgender/Non-Binary/Different Identity	0.4% (2)	1% (7)
**Race/Ethnicity** ^**16**^ **^**	**% (n)**	**% (n)**
Underrepresented minority in STEM/Medicine	14.4% (60)	15.4% (116)
White	68% (263)	66.6% (477)
Asian	15% (58)	16.5% (118)
**Socioeconomic Disadvantaged background** ^**17**^	**% (n)**	**% (n)**
Yes to 2+ criteria	20.3% (74)	16.5% (112)
No	75% (273)	78.4% (534)

* There was between a 10-15% non-response rate for demographic questions in the membership form.

^ some data categories have been collapsed for streamlined reporting.

# Some members have multiple institutional appointments/affiliations.

### Implementation of continuous data collection forms (consultation & event form)

Several individuals were involved in building the EP. One 0.20 FTE faculty advisor worked with Cores to develop an evaluation plan, create initial logic models, conceptualize data collection instruments, inventory extant data, and design the evaluation platform. One 1.0 full-time equivalent (FTE) Evaluation Director utilized their full-time effort over 4 months to finish designing and implementing the continuous data collection instruments in REDCap. Informatics faculty contributed effort to dashboard design. A programmer contributed effort to build the dashboard based on an existing model. The Evaluation Director, 0.20 FTE faculty advisor, the REDCap administrator, and dashboard programmer participated in weekly meetings to troubleshoot building of the forms and dashboard. Once implemented, ongoing maintenance of evaluation activities and the platform has been achieved through 0.125 FTE spread across an evaluation director and a program assistant. The consultation and event forms were completed in November 2022. A soft launch of the forms occurred during November and December 2022 to test its flow and functionality while also preparing training materials for the users. The evaluation team conducted data quality checks with test records to ensure that alert coding was accurate and data transfer between membership module and other REDCap instruments was accurate.

Beginning in January 2023, the evaluation team held several education and training meetings with Navigators from each core to instruct them on how to use the REDCap system and how to respond to email alerts. Uptake was slow and required repeated training sessions and one-on-one consultation sessions to reinforce the flow of the consultation form system. However, during this time, the team made iterative changes based on feedback to align with the Navigators’ workflow and needs. When first implemented, Navigators averaged over 14 days to respond to and address consult requests. After a year of implementation, the average time is now less than 7 days.

The Evaluation team lead continues to attend the Navigators monthly meetings to address continuous educational needs and there is a standing agenda item to discuss any EP changes and continuous improvement and quality needs. EP training has also been added to the Navigators onboarding checklist and new Navigators are given one-on-one training with the Evaluation team to familiarize themselves with the EP and their responsibilities. Table 2 demonstrates the Navigators’ and Members’ completion rates and workflow for the first two years of implementation.

**Table 2.** Form completion rates for fiscal years 2023 & 2024

	FY 2023	FY 2024	Total N (% completion)
Total Consultation Requests	109	172	281
Total Consultations Scheduled	107	167	274
Total Consultations Occurred	103	152	255 (93%)
Satisfaction Surveys Completed*	28	31	59 (46%)
Average Consultation Satisfaction Score^^^	4.3	4.5	4.4
Long-term outcomes Surveys Completed^#^	N/A	22 (20%)	22 (20%)

* If Navigators were back-entering data, they could choose to opt-out of sending a satisfaction survey to reduce confusion.

^ On a 5-point likert scale of 1-Strongly Disagree to 5- Strongly Agree.

# *n* = 110 due to consults recorded up to June 30, 2023 would have long term survey been sent by June 30, 2024.

### Annual data collection instruments

In July 2023, the first round of the Projects Outcomes and Impacts survey was deployed. The evaluation team spent the prior few months consolidating old data about past pilot awardees into one central database to understand the available baseline data. Due to high staff turnover at the CTSI and the restructuring of the pilot awards program, documentation was scattered. Once this information was collected, it was uploaded into REDCap to serve as the baseline database for long-term outcomes and impacts tracking.

The team deployed the initial survey to investigators who received a Frontiers award prior to 2020. The decision was based on the idea that by 2023, awardees were two years removed from their funding end date and a year removed from the last outcomes reporting related to the RPPR. Surveys were sent to 181 awardees whose current email addresses were readily available. We achieved a partial response rate of 40%, however, of those who responded, only 24% completed all forms of the survey.

In August 2023, the initial member assessment was deployed to all registered Frontiers members (*n* = 419). We achieved a response rate of 22%. While individual responses were anonymous, we were able to determine the aggregate demographic make-up of responders, and they closely aligned with the overall membership demographics at that time. The majority (66.3%) of respondents had been engaged with the Frontiers network for the past 1–5 years and 55% has utilized a Frontiers service in the past 12 months. Respondents indicated that they felt the level of communication from Frontiers was “just right” and got their updates from Frontiers newsletter, rather than social media. Overall, respondents felt their Frontiers involvement enhanced their translational science knowledge, facilitated collaborations, and advanced their career. Respondents identified gaps in awareness of services and resources. Results supported administrators’ identification of these issues and provided further justifications for planned interventions. An infographic was developed to communicate responsiveness to investigators’ needs that was shared with the entire Frontiers membership: https://frontiersctsi.org/doccenter/dfd4efdc98be455a9cf4f2165df0282c

### Dashboard

We launched the first iteration of the Evaluation Dashboard in Summer 2023. Navigators reviewed a beta version during a monthly meeting to provide feedback on the layout and content that would be most useful for their reporting. After final edits, the link to the dashboard was distributed to all Frontiers support staff. At the time of publication, the dashboard is only available to Frontiers staff for internal reporting purposes. We plan to expand the dashboard to be public facing, but there is a need to refine and add context to the reported data before public consumption.

### Reporting

In the interest of data transparency, biannual overview evaluation reports are given to the Frontiers Operations group, which consists of all faculty and staff involved in the CTSI’s operations (*n* ∼ 93). Additionally, the Director of Evaluation makes quarterly reports on progress to the Stakeholder Advisory Committee, which consists of representatives from each of the partner institutions formally involved with Frontiers. Each December, a Core report is generated to update each Core on Logic Model activity progress and link those activities to outputs that have been captured in the EP.

## Discussion

Several aspects of the EP development and implementation process have more closely aligned the institute with best evaluation practice and CTSA standards. The continued utilization of logic models throughout the programmatic activities is in line with best practice [15]. Regular review of activities has allowed Cores to reassess feasibility of activities, identify upcoming priorities, and focus attention toward poorly progressing activities. It has provided a series of checks and balances between the CTSI’s leadership and Core leads on their expectations and the supports that are needed for each Core. Logic Model reviews have provided Cores with tools to continuously monitor and communicate their progress and have improved communication between the evaluation team and programmatic team to reevaluate the data gaps in evaluation efforts and refine the evaluation workflow.

Consistent with the UFE framework, our build and implementation reinforce our leadership’s commitment to communication, continuous improvement, and transparency. The alignment of variables and their definitions such as career stages [16], race, ethnicity [17], and disadvantaged status [18] in the membership form are an example of improved data efficiency and quality by creating standardized demographic categories used across the institute and requiring members to only submit these data upon registration for membership. Evaluation reports delivered to each core for their preparation of annual progress reporting sections have reduced inconsistencies in data across sections and reduced the time burden for completion of annual reports. The evaluation dashboard is an additional component of the team’s data transparency focus and provides real-time output data across all the program areas within Frontiers and gives internal staff some agency over accessing and reviewing their program’s outputs and outcomes.

Collecting membership demographic information upfront also allows for more robust data analysis. Many other hubs utilizing the membership model did not initially collect member demographics and now rely on retrospective data collection efforts that engender missing data [19]. The membership model ensures complete data, allows better understanding of the composition of Frontiers’ network, and allows tailoring of communication according to members’ preferences. In the age of overcommunication and marketing emails, a tailored communication approach can improve engagement in the institutes activities [20] with a targeted advertisement for certain activities and resources.

The evaluation team participates in national CTSA workgroups, ensuring that collected demographics are in alignment with other hubs. In turn, by selecting the REDCap platform, the evaluation process is accessible to all Frontiers partner institutions. Survey forms are shareable between institutional instances of REDcap and can be obtained by contacting the corresponding author. Since REDCap is a free, PHI-compliant data collection, management, and survey platform [9,10], its adoption did not require an extensive financial or educational investment. Institutional experts and administrators were also in place to assist with troubleshooting the platform and requesting customization.

While development and implementation took initial investment, since implementation, maintenance effort has been modest. In the context of a five-year grant’s operations, minimal dedicated FTE was needed to develop, implement, and maintain the platform. Based on ongoing user feedback and documentation of Navigator activities, the implementation of the EP has improved the workflow of Frontiers operations. It has allowed data collection and tracking to become centralized and has created a consensus on variables of interest and utilized best practices and national standards on data language. In one-on-one conversations with Navigators, they have reported personal increased knowledge of their roles related to the implementation of the EP and appreciate having a systematic way to track their workflow. Table 2 also demonstrates highly efficient completion rates of the consultation forms by the navigators suggesting that this process has experienced high internal uptake as well as minimal administrative burden on the users. Our satisfaction survey response rates (46%) are also in line with research survey response rates (44%) [21].

Moreover, the development of the Frontiers EP has created a multi-level approach to impact assessment within the CTSI. With the logic models serving as the framework for activities and outputs, we were able to identify which outcomes need to be assessed at an individual and organizational level. The goal of CTSAs is to provide infrastructure to the research enterprise to conduct translational science, and a multi-level assessment of outcomes provides the most robust view of their impact. Creating this platform to capture the programmatic outputs allows the leadership and Cores to get a macro-level understanding of their activities’ impact and allows for continuous improvement. Because of the centralized platform, we can compare outcomes over time and assess where programmatic areas have made improvements and where support is still needed.

Additionally, with the creation of the platform, logic models, and data flow outlines, it has allowed us to map our activities and outputs specific to concepts of the Translational Science Principles [22,23]. and Translational Sciences Benefits Model (TSBM) [24]. Within the logic models, we have identified TSPs the activity addresses and the data in the EP that provides evidence of this translational science activity. The TSBMs are integrated into the EP at longitudinal data collection points such as Consultation Outcomes surveys and Annual Project Outcomes surveys. Again, with these surveys being centralized and standardized within our CTSA, we can comprehensively capture and demonstrate how our hubs activities are effectively addressing and impacting translational science and health.

Finally, with the sunsetting of the Common Metrics Initiative [25,26], expectations on what data collection was relevant to local and national stakeholders were unclear. The Common Metrics focused on specific outputs of program areas but failed to capture the true breadth of a CTSA’s work and impact [27]. The Frontiers EP helps fill those gaps by truly mapping each programmatic area with a specific data collection variable, creating a comprehensive approach to the CTSI’s evaluation.

### Limitations and next steps

While REDCap is a powerful and widely utilized platform, complaints from external users have noted that the interface can be clunky, and some functionalities are not user-friendly compared to other software programs. We have addressed user design and flow by making branching and flow changes as requested, however, not all suggestions can be accommodated due to REDCap’s functionalities. Some features still require manual manipulation to extract the appropriate data from relevant projects and to link it to the appropriate dataset. This requires about 5 hours per week of data entry from the evaluation team. Data analysis within REDCap is also limited, creating added steps of pulling down data to clean and manipulate variables before uploading to analytic software for in-depth analysis. We have tried to address this through the dashboard development with the ability to filter data based on time and additional variables to provide year and quarterly comparisons on metrics.

Staff capacity may also be a limitation in the feasibility of widespread implementation of such a platform. Based on communication with other hubs, the level of collaboration between Evaluation teams and their REDCap support/Informatics team varies widely. A major contributor to the success of this implementation was the close working relationship between the Evaluation Lead, REDCap Administrator and Faculty Advisor during the building and implementation process. The Evaluation Lead did the primary REDCap building with the Administrator providing technical programing support and troubleshooting along the way and with the Faculty Advisor providing the conceptual framework for the platform. The initial buy-in from leadership in the development of this platform was crucial for ensuring dedicated staff and faculty effort to implement this platform effectively.

With the breadth of the data being collected in alignment with logic model outcomes, we have not yet developed an automated way to directly connect these two pieces. The team is exploring the utility of PowerBI to potentially integrate the logic models directly with the platform so data outputs captured in REDCap can be directly connected to the stated activities within the logic model. And as with all new platforms, systems, and workflows, continued human investment and engagement are needed to sustain the platform. The utilization of multiple staff and leadership champions is critical for the sustainability of the platform and to protect against potential staff turnover [28].

### Next steps

Enhancements to the EP are ongoing and the team will continue to work with Frontiers members to make necessary adjustments to improve user experience and optimize workflow. We continue to update the reporting process through the Evaluation Dashboard and regular required reports. While the dashboard is only available to internal Frontiers staff, with additional context and formatting, we plan to make the dashboard publicly available. As of January 2025, work is underway to make the dashboard accessible through affiliate accounts at partner institutions. This will provide an opportunity for further investigation of how data transparency and feedback prompt behavior and programing change within Frontiers. Reports will also be developed from the EP in 2025 to help with initial grant renewal preparations.

## Conclusion

Previous efforts to evaluate CTSAs have focused on individual programmatic and global impact: mentorship programs [29], early career training programs [30], and pilot funding programs [31] through the use of case studies [4] and bibliometrics [1]. Yet many core functions of the CTSAs such as consulting services, workforce training, and relationship building have been harder to capture without consistent and standardized data collection to capture more granular processes. Further, few efforts have captured the quality of services provided to truly understand the independent impact of the CTSA itself. With additional scrutiny of federal initiatives, platforms such as the Frontiers EP may be necessary to demonstrate impact. We believe this evaluation platform can be a viable solution for not only other CTSA hubs but also other expansive research institutes to capitalize on already established resources within their institutions. Our utilization of REDCap, centralization, and standardization of data capture, adaptability, and integration of user input and refinement makes this platform a tool that can be utilized in different clinical and translational science contexts.

## Supporting information

10.1017/cts.2025.10047.sm001 Padek et al. supplementary material Padek et al. supplementary material

## Supplementary material

For supplementary material accompanying this paper visit https://doi.org/10.1017/cts.2025.10047
