# Supplementary material for: Building an evaluation infrastructure to capture process and progress within a Clinical and Translational Science Awards hub
Source: J Clin Transl Sci. 2025 Jun 9;9(1):e136. doi: 10.1017/cts.2025.10047 (PMC12260981; doi:10.1017/cts.2025.10047)
Supplement: Padek et al. supplementary material [file S2059866125100472sup001.pdf]

Become A Member

Please complete the survey below.

Your membership in Frontiers is linked through your email address. If you have previously registered as a member, you will recieve a pop-up alert that indicates "Duplicate Value." You do not need to continue completing the form if you recieve that alert.

Thank you!

Registration date ____________________

EMAIL ADDRESS (Your email address will be your unique identifier for all Frontiers services) ____________________
(If not using a business email address, access to documents may be limited.)

First name ____________________

Last Name ____________________

Full Name ____________________

ADDRESS ____________________

ADDRESS LINE 2 ____________________

CITY ____________________

STATE ____________________

ZIP CODE ____________________

PHONE NUMBER ____________________

Twitter handle ____________________

eRA Commons User Name ____________________
(For researchers)

ORCID ID ____________________

Role	◯ Community Member<br>◯ Faculty/Clinicians<br>◯ Trainee/PostDoc<br>◯ Public/Private Sector<br>◯ Other<br>(deprecated field)
OTHER AFFILIATION	__________________________________<br>(deprecated field)
Frontiers Role	☐ TL1/KL2 Scholar<br>☐ TL1/KL2 Mentor<br>☐ Frontiers FTE<br>☐ Pilot awardee
INSTITUTION	☐ CHILDREN'S MERCY KANSAS CITY<br>☐ KANSAS CITY UNIVERSITY<br>☐ KANSAS STATE UNIVERSITY<br>☐ KANSAS UNIVERSITY MEDICAL CENTER, KANSAS CITY<br>☐ KANSAS UNIVERSITY MEDICAL CENTER, SALINA<br>☐ KANSAS UNIVERSITY MEDICAL CENTER, WICHITA<br>☐ SAINT LUKE'S<br>☐ THE UNIVERSITY OF KANSAS HEALTH SYSTEM<br>☐ UNIVERSITY OF KANSAS, LAWRENCE<br>☐ UNIVERSITY OF MISSOURI, KANSAS CITY<br>☐ COMMUNITY ORGANIZATION<br>☐ PRIVATE/INDUSTRY ORGANIZATION<br>☐ OTHER
COMMUNITY ORGANIZATION	◯ JUNTOS<br>◯ UZAZI VILLAGE<br>◯ DOWN SYNDROME INNOVATIONS<br>◯ RAREKC<br>◯ NBC COMMUNITY DEVELOPMENT CORP.<br>◯ LIVEWELL FINNEY COUNTRY<br>◯ COMMUNITY HEALTH COUNCIL OF WYANDOTTE COUNTY<br>◯ MASONIC CANCER ALLIANCE<br>◯ WYANDOTTE COUNTY HEALTH EQUITY TASKFORCE<br>◯ OTHER
PRIVATE ORGANIZATION	__________________________________
ARE YOU THE RECIPIENT OF A PAST FRONTIERS AWARD?	◯ Yes<br>◯ No
HAVE YOU EVER RECEIVED EXTERNAL GRANT FUNDING?	◯ Yes<br>◯ No
PRIMARY AREA OF RESEARCH OR TRAINING	◯ PRE-CLINICAL RESEARCH<br>◯ CLINICAL RESEARCH<br>◯ IMPLEMENTATION RESEARCH<br>◯ POPULATION-BASED RESEARCH<br>◯ BASIC RESEARCH<br>◯ NA
PLEASE ADD UP TO 5 KEYWORDS DESCRIBING YOUR RESEARCH	__________________________________

Keyword 2	________________________
Keyword 3	________________________
Keyword 4	________________________
Keyword 5	________________________
WHY ARE YOU INTERESTED IN BECOMING A MEMBER?	☐ UTILIZING RESOURCES<br>☐ SEEKING TRAINING<br>☐ SEEKING MENTORSHIP<br>☐ SEEKING COLLABORATORS<br>☐ SEEKING FUNDING<br>☐ DEVELOPING NOVEL METHODS<br>☐ PROVIDING RESOURCES<br>☐ PROVIDING TRAINING OR MENTORING<br>☐ SEEKING RESEARCH SUBJECTS<br>☐ SEEKING PARTNERSHIP FOR MY COMMUNITY<br>☐ OTHER
FOR WHAT OTHER REASON ARE YOU INTERESTED IN BECOMING A MEMBER OF FRONTIERS?	________________________
IN WHAT WAYS ARE YOU WILLING TO PARTICIPATE?	☐ MENTOR FOR TRAINEES<br>☐ PROTOCOL REVIEW COMMITTEE MEMBER<br>☐ GRANT REVIEW COMMITTEE<br>☐ SHARE EXPERTISE/RESOURCES<br>☐ SERVE ON A PANEL/OTHER COMMITTEE<br>☐ SPEAKER<br>☐ PATIENT OR COMMUNITY ADVISORY BOARD<br>☐ MOCK STUDY SECTION<br>☐ TRAINEE<br>☐ OTHER
IN WHAT OTHER WAYS YOU ARE WILLING TO PARTICIPATE?	________________________
WHAT TYPES OF INFORMATION WOULD YOU LIKE TO RECEIVE FROM FRONTIERS?<br><br>You can opt-out of specific communication by unchecking the boxes.	☐ NEWSLETTER<br>☐ DISCOVERY AND RESEARCH GRAND ROUNDS<br>☐ SEMINARS/WEBINARS<br>☐ FUNDING<br>☐ MOCK STUDY SECTION<br>☐ GENERAL NEWS/UPDATES<br>☐ UPDATES/NEWS FROM FRONTIERS PARTNERS<br>☐ CTSA NEWS<br>☐ OTHER
WHAT OTHER INFORMATION YOU WOULD LIKE TO RECEIVE?	________________________

Demographics

Frontiers is committed to supporting a diverse clinical and translational science workforce. Please provide the following information so we can track our progress.

We aim to be inclusive but want to protect your privacy. Data will be stored in a REDCap database. This data will be available to the Frontiers evaluation team. It will not be shared with navigators fulfilling your request or peer reviewers. Data may be reported to Frontiers PIs, Core Leaders, Partner Institution Administrators, and the NIH, but only in the aggregate. Individually identifiable data will not be shared. We will follow research best practices in reporting data for categories with small cell sizes.

Responses to these questions are optional. This data is only reported in the aggregate and individual responses are kept confidential. Frontiers utilizes this information to better understand the make-up of our membership and how we can tailor programs and services to best fit everyone's needs.

What is your current career stage?

- ◯ Experienced investigator Investigators who have competed successfully as a PI to receive substantial independent NIH funding
- ◯ New investigator Investigators who have not yet competed successfully as a PI to receive substantial independent NIH funding
- ◯ Post-doctoral investigator
- ◯ Graduate student trainee
- ◯ Resident or Fellow
- ◯ Medical student trainee
- ◯ Undergraduate student
- ◯ Research Staff ______
- ◯ Other ______

Do you identify as Hispanic or Latino?

A person of Cuban, Mexican, Puerto Rican, South or Central American, or other Spanish culture or origin regardless of race

- ◯ Yes
- ◯ No
- ◯ Prefer not to say

What is your race? (Check all that apply)

- ☐ American Indian or Alaska Native
- ☐ Asian
- ☐ Black or African American
- ☐ Native Hawaiian or Other Pacific Islander
- ☐ White
- ☐ Different identity: ______
- ☐ Prefer not to say

How would you describe your gender identity? (Check any that apply)

- ☐ Woman
- ☐ Man
- ☐ Transgender
- ☐ Non-Binary/Non-conforming
- ☐ I don't see an option that describes me
- ☐ Different Identity
- ☐ Prefer not to respond

Additional Gender Identities

- ◯ agender
- ◯ androgyne
- ◯ demigender
- ◯ genderqueer or gender fluid
- ◯ Two Spirit
- ◯ questioning or unsure
- ◯ additional gender category/identity ______

Do you think of yourself as (Check one):	◯ Straight or heterosexual<br>◯ Lesbian, gay or homosexual<br>◯ Bisexual<br>◯ Queer<br>◯ Don't Know<br>◯ Prefer to self-describe ______<br>◯ Choose not to disclose
Do you have a physical or mental impairment that substantially limits one or more major life activities, as described in the Americans with Disabilities Act?	◯ Yes<br>◯ No<br>◯ Never<br>◯ Sometimes<br>◯ Often<br>◯ Always<br>◯ Prefer not to say
Do you meet two or more of the criteria for disadvantaged status?<br>Criteria (below) updated by NIH in 11/2019Two or more of the following: Were or currently are homeless, as defined by the McKinney-Vento Homeless Assistance Act (Definition)Were or currently are in the foster care system, as defined by the Administration for Children and Families (Definition)Were eligible for the Federal Free and Reduced Lunch Program for two or more years (Definition)Have/had no parents or legal guardians who completed a bachelor's degree (see link)Were or currently are eligible for Federal Pell grants (Definition)Received support from the Special Supplemental Nutrition Program for Women, Infants and Children (WIC) as a parent or child (Definition)Grew up in one of the following areas: a) a U.S. rural area, as designated by the Health Resources and Services Administration (HRSA) Rural Health Grants Eligibility Analyzer, or b) a Centers for Medicare and Medicaid Services-designated Low-Income and Health Professional Shortage Areas (qualifying zipcodes are included in the file). Only one of the two possibilities in #7 can be used as a criterion for the disadvantaged background definition.	◯ Yes<br>◯ No<br>◯ Prefer not to say
Have you ever served on active duty in the U.S. Armed Forces, military Reserves, or National Guard?	◯ Yes<br>◯ No
